# Effect of “Jian-Pi-Zhi-Dong Decoction” on Gamma-Aminobutyric Acid in a Mouse Model of Tourette Syndrome

**DOI:** 10.1155/2014/407509

**Published:** 2014-04-09

**Authors:** Wen Zhang, Wenjing Yu, Daohan Wang, Li Wei, Minkyoung Lee, Sumei Wang

**Affiliations:** ^1^Department of Pediatrics, Dongfang Hospital, Beijing University of Traditional Chinese Medicine, Beijing 100078, China; ^2^School of Nursing, Taishan Medical University, Taian 271016, China

## Abstract

The purpose of this study was to explore the positive effects of Jian-Pi-Zhi-Dong Decoction (JPZDD) on Tourette syndrome (TS) by investigating the expression of gamma-aminobutyric acid (GABA) and its type A receptor (GABA_A_R) in the striatum of a TS mice model. The model was induced by 3,3′-iminodipropionitrile (IDPN) treatment; then mice were divided into 4 groups (n=22, each); control and IDPN groups were gavaged with saline and the remaining 2 groups were gavaged with tiapride and JPZDD. We recorded the stereotypic behaviors of TS mice and measured the content of GABA in striatum by HPLC and GABA_A_R expression by immunohistochemistry and real-time PCR. Our results showed that JPZDD inhibited the abnormal behaviors of TS model mice and decreased GABA levels and GABA_A_R protein and mRNA expression in the striatum of TS model mice. In brief, the mechanism by which JPZDD alleviates TS symptoms may be associated with GABA_A_R expression downregulation in striatum which may regulate GABA metabolism.

## 1. Introduction

Tourette syndrome (TS) is a developmental neurobehavioral disorder characterized by stereotypic, involuntary, repetitive movements and noises, referred to as chronic (more than one year) motor and vocal tics, respectively [1]. TS usually starts in childhood and tends to peak at onset of puberty and improve by the end of adolescence [2]. In addition, TS patients also show behavioral and emotional disturbances including symptoms of attention deficit hyperactivity disorder (ADHD), obsessive compulsive disorder (OCD), and pervasive developmental disorder [3].

Until now, the etiological and pathophysiological mechanism of TS remained unclear. It is generally agreed that the basal ganglia, including circuits that link the striatum to cortex, are dysfunctional [4]. The basal ganglia form a network of interconnected subcortical structures, including the striatum, globus pallidus, substantia nigra, and subthalamic nucleus. In the cortico-striato-thalamocortical (CSTC) circuit, the cerebral cortex provides the main input to the striatum; then the striatum relays information to the globus pallidus and substantia nigra through two pathways: one excitatory (direct pathway) and one inhibitory (indirect pathway). Malfunction of these circuits may contribute to the behaviors that manifest as tics. For a long time, abnormal dopaminergic activity in the synaptic cleft has been considered the most important factor in TS [1]. Most scholars postulate that hyperactivity of dopaminergic neurons and/or dopamine receptors underlie the pathophysiology of TS [5–7]. However, the results obtained in these previous studies were inconclusive. Both DA and GABAergic neurons are crucial for the maintenance of basal ganglia circuits. Therefore, other imbalances, such as in glutamatergic or GABAergic metabolism, seem probable in TS [8]. Recently, it was proposed that disrupted excitatory/inhibitory balance in key circuits might underlie many neurodevelopmental disorders including TS [9]. Dopaminergic and GABAergic systems interact in the basal ganglia system; GABAergic neurons in corticostriatal pathways have been reported to either directly or indirectly activate the dopaminergic system [10]. Clinical studies also showed that metabolic disturbance of GABA was involved in the pathogenesis of TS [11, 12].

The amino acid neurotransmitter GABA is the principal inhibitory neurotransmitter in the central nervous system (CNS). GABA regulates brain excitability via its GABA_A_ receptors [13]. GABA receptors are key targets for drug design to treat various tics, and GABA_A_ receptor systems may play a major role in the pathophysiology of TS [14]. Levetiracetam, with a typical enhancing activity at GABA_A_ receptors, could be of interest for treatment of tics [15]. GABA_A_ receptors are composed of five subunits that together comprise more than 19 different classes, including *α*1–6, *β*1–3, *γ*1–3, *δ*, *ε*, *θ*, *π*, and *ρ*1–3 [16]. The GABA_A_ alpha 4 receptor was predicted to be spliced in the pathophysiology of TS and tics [12].

A previous study has proven that Jian-Pi-Zhi-Dong Decoction (JPZDD) can effectively inhibit abnormal behavior in a TS mouse model and increase the level of dopamine transporter in the striatum [17]. However, this study did not explain the phenomenon of higher expression of DAT in some TS patients and does not completely explain the pathophysiology. We therefore hypothesized that an abnormality in the function of CSTC circuits could also be caused by pathological changes in GABA and its receptors. The present study aimed to explore the possible mechanism of JPZDD on the GABAergic system of a TS mouse model, in particular the GABA_A_ receptor.

## 2. Materials and Methods

### 2.1. Drugs and Reagents

3,3′-Iminodipropionitrile (IDPN) was purchased from Sigma-Aldrich Co., LLC. (St. Louis, MO, USA), and tiapride (Tia) from Jiangsu Nhwa Pharmaceutical Co., Ltd. (Xuzhou, Jiangsu, China). Gamma-aminobutyric acid (GABA, 5835) and o-phthaldialdehyde (OPA, P0657) were purchased from Sigma, beta-mercaptoethanol (*β*ME, 0482) from Amresco Co., LLC. (Solon, OH, USA), anti-GABA _A_ receptor alpha 4 antibody (primary antibody, 1 : 100) from Abcam Co., LLC. (Hong Kong, China), and poly-HRP anti-rabbit IgG secondary antibody (PV-9001) from ZSGB-BIO Co. (Beijing, China).

### 2.2. Preparation of JPZDD

Ten different Chinese medicinal herbs were included in the JPZDD. They were purchased from the Pharmaceutical Department at Dongfang Hospital affiliated to Beijing University of Chinese Medicine (BUCM). Director Qing-chun Hao identified components, and the voucher specimens were deposited. JPZDD contains ten ingredients: 10 g* Pseudostellaria heterophylla*, 10 g* Atractylodes macrocephala* Koidz, 10 g* Poria cocos* Wolf, 6 g* Pinellia ternata* Breit, 6 g* Citrus reticulata* Blanco, 6 g* Saposhnikovia divaricata* Schischk, 3 g* Gentiana scabra* Bge, 10 g* Angelica sinensis* Diels, 6 g* Ligusticum chuanxiong* Hort, and 10 g* Uncaria rhynchopylla* Jacks. All herbs were soaked for 1 h at room temperature and decocted with distilled water for 2 h. The filtrate was condensed and dried at 60°C using a vacuum-desiccator. The extracted granules were analyzed by infrared fingerprint compared with standards to guarantee a qualified rate of more than 90% and then packaged and stored at room temperature.

### 2.3. Animals

We used eighty-eight male ICR mice, weighing 18 ± 2 g, aged 4 weeks, purchased from Vital Rive Laboratories, Beijing, China (no. SCXK 2012-0001). All animal experimental protocols conformed to the Animal Management Rules of the Chinese Ministry of Health, and the study was approved by the Animal Ethics Committee of the Chinese Academy of Medical Sciences. Animals were kept in a standard animal feeding room with a room temperature of 21 ± 1°C and relative humidity of 30% to 40%, on a 12 h light-dark cycle (light for 12 h: 07:00 to 19:00 and darkness for 12 h: 19:00 to 07:00) and ad libitum access to purified water. The mice were fed for 1 w before generating the TS model. After one week, the mice were randomly divided into a saline group (control group, *n* = 22) and a TS model group (*n* = 66). The saline group was injected (i.p.) with 0.9% saline (15 mL kg^−1^); the TS model group was injected (i.p.) with IDPN (350 mg kg^−1^) once a day for 7 consecutive days. According to the behavioral measurements used for evaluating grades of stereotype, we made behavioral recording of the TS model group, as described by Wang et al. [17]. The TS mouse model group was further divided into 3 groups: IDPN model group (*n* = 22), IDPN+Tia group (*n* = 22), and IDPN+JPZDD group (*n* = 22). The saline and IDPN group were gavaged with saline (0.9%) at 20 mL Kg^−1^, the IDPN +Tia group with Tia at 50 mg kg^−1^, and the IDPN+JPZDD group with JPZDD at 20 g kg^−1^ once a day for 6 consecutive weeks. Behavioral recordings were conducted by 2 trained observers who were familiar with the measurements but blind to the group condition. Each animal was observed for 2 min [18], and the average score was calculated. On the last day of the trial, the mice were euthanized by cervical dislocation.

### 2.4. High Performance Liquid Chromatography (HPLC)

The dorsolateral striatum from both the left and right sides (*n* = 10, per group) was dissected and frozen at −80°C. Brain tissue samples were weighed and then homogenized in 700 *μ*L of ice-cold lysis buffer [19], containing o-phthalaldehyde 27 mg, anhydrous ethanol 1 mL, tetraborate buffer 9 mL, and**β**-mercaptoethanol 5 *μ*L.

The homogenate was centrifuged at 14000 rpm for 15 min at 4°C and filtered through a 0.22 *μ*m filter (Costar, Spin-X) and then centrifuged at 7000 rpm. Standard solution or the filtrate obtained from brain homogenate (20 *μ*L) was injected into the HPLC. Chromatographic conditions were as follows. The precolumn was Shiseido (Guard Cartridge, Capcell C18 MG S-5, 4.0 × 10 mm). The chromatographic column was Waters XTerra MS (3.0 × 50 mm, 2.5 um, Part no. 186000598). The mobile phase was composed of 100 mM disodium hydrogen phosphate, 25% methanol, and 10% acetonitrile (pH 6.70). The flow rate was 0.6 mL/min. The column oven was held constant at 40°C. Working solutions of GABA (40, 20, 8, 4, 2, 1, and 0.5 *μ*g/mL) were used. The calibration curve of each compound was determined by plotting the ratio of peak area to internal standard versus concentration of the spiked standard solution. A linear regression equation (*y* = *ax* + *b*) was evaluated, where *x* is the concentration of the analytes and *y* is the peak area ratio. The correlation coefficient (*R* ^2^) was calculated.

### 2.5. Immunohistochemistry

Mice (*n* = 6, per group) were perfused and fixed with 4% PFA after anesthetizing with 10% chloral hydrate. The brains were removed and postfixed in 4% PFA for 14 h and then embedded in paraffin. The embedded brains were cut into 3 *μ*m sections. After dewaxing, rehydration, and blocking, the sections were incubated with an alpha 4 primary antibody (ab4120, Abcam, Hong Kong) at a dilution of 1 : 100 overnight at 4°C [20]. After washing with PBS three times, the tissues were incubated with secondary antibody (PV-9001, ZSGB-BIO, China) for 30 minutes. Sections were visualized by incubating with by DAB (1 : 20) for 5 min and then coverslipped using neutral balsam. Six visual fields were chosen randomly from bilateral stained striatum under an upright microscope at 20 x magnification. The optical density (OD) of each field was calculated using the Image-Pro Plus 6.0 analysis system. The values of GABA_A_ receptor alpha 4 protein in the striatum were measured in every three brain slices of each mouse.

### 2.6. Real-Time PCR

The dorsolateral striatum from both the left and right sides (*n* = 6, per group) was dissected and frozen at −80°C. Total RNA from the striatum was isolated with trizol reagent according to the manufacturer's protocol (Tiangen Biotech, Beijing, China). GABA alpha 4 primers were as follows: forward primer: 5′-GCTGACAGAGGGAAATAAATAAAG-3′ and reverse primer 5′ -TGGATGATTCTGGTAGAGTGGG- 3′. Beta actin primers were as follows: forward primer 5′ -GCCTTCCTTCTTGGGTATGGAA- 3′ and reverse primer 5′ -CAGCTCAGTAACAGTCCGCC- 3′. The basic protocol for real-time PCR was an initial denaturation at 95°C for 10 min, followed by 45 cycles of amplification. For cDNA amplification, the cycles consisted of denaturation at 95°C for 10 min, annealing at 95°C for 15 s, and elongation at 60°C for 60 s. The SYBR green signal was detected using IQ5 real-time PCR machine (Bio-Rad, Hercules, CA, USA). PCR products were analyzed by gel electrophoresis and melting curve analysis to confirm specific amplifications. mRNA expressions were normalized using *β*-actin. Transcript levels were quantified using the ΔΔ*Ct *value method.

### 2.7. Statistical Analysis

Results were expressed as the mean ± S.E.M. The significance of differences was examined using ANOVA, followed by Student-Newman-Keuls (SNK) test. In all tests, the criterion for statistical significance was *P* < 0.05.

## 3. Results

### 3.1. Behavior Study

**Figure 1:**
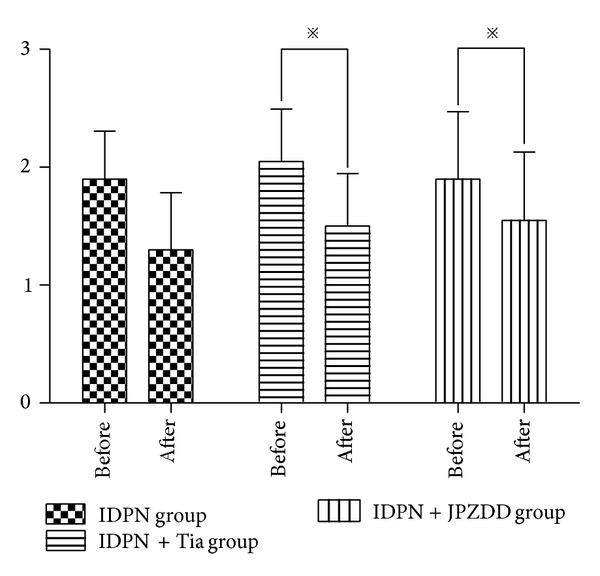
Evaluations of stereotypy of TS mice model in different groups before and after the 6 weeks of treatment. The stereotypy score decreased in all groups, but a significant effect of treatment was observed in the IDPN + Tia group and the IDPN + JPZDD group. Data were shown as mean ± SEM (*n* = 22 mice/group); *※* represents *P* values <0.05.

IDPN could induce stereotyped behavior significantly without intervention compared with the saline control group. TS mouse model induced by IDPN showed abnormal stereotypes in different degrees. After the IDPN injection, we divided the TS mouse model into three groups according to the score to ensure that there was no statistical significance among these groups, and then we gave the different treatment. The IDPN + JPZDD and IDPN + Tia groups showed a significant decrease in severity of stereotyped behavior before and after treatment (*P* < 0.05), but the score of stereotypes for the IDPN group had no statistical significance (*P* > 0.05) (Figure 1).

### 3.2. Content of GABA in Striatum by HPLC

**Figure 2:**
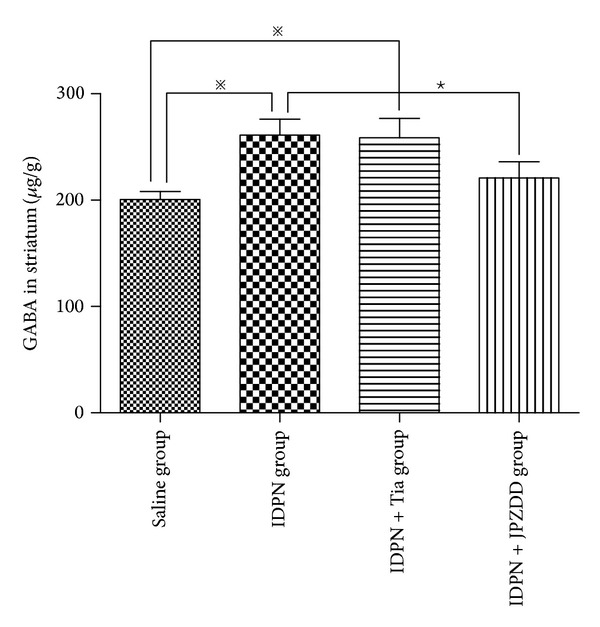
HPLC to evaluate the content of GABA in striatum. The bar graphs represent data from 40 different animals. Data were shown as mean ± SEM (*n* = 10 mice/group). *※* represents *P* values <0.05; ★ represents *P* values <0.01.

Content of GABA in striatum was measured by HPLC. At the end of the treatment, the level of GABA in the IDPN group (261.24 ± 47.31) and IDPN+Tia group (258.57 ± 57.64) was significantly higher than the saline group (200.54 ± 23.94) (*P* < 0.05). The content of GABA in the IDPN+JPZDD group (220.90 ± 48.30) was lower than the IDPN group (*P* < 0.01) (Figure 2).

### 3.3. Level of GABA_A_R in Striatum by IHC

**Figure 3:**
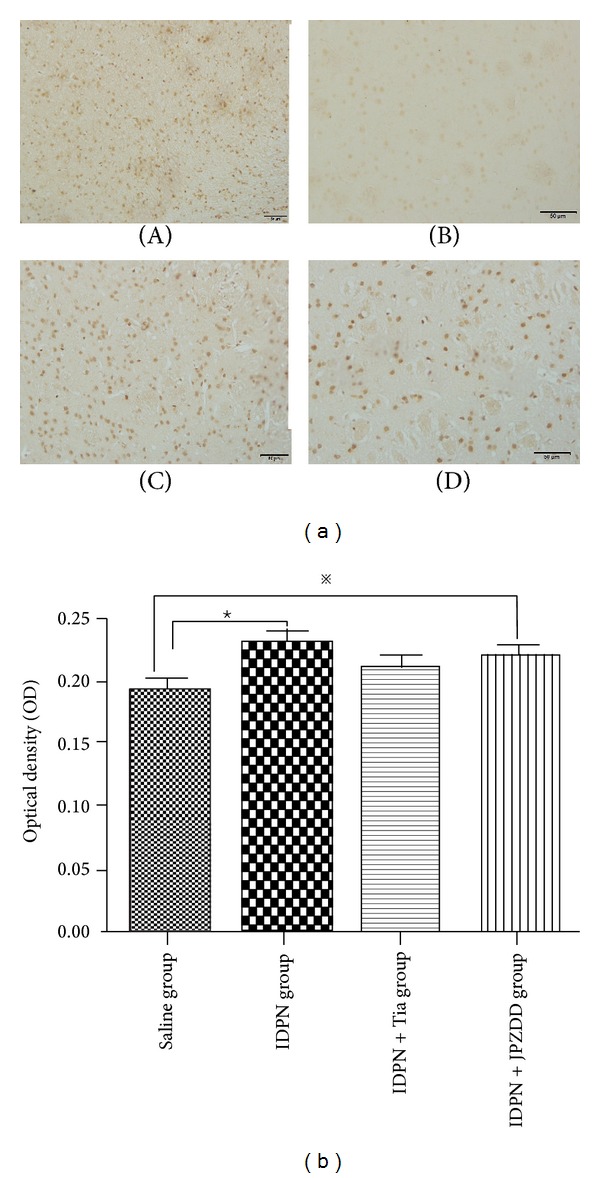
Immunohistochemistry of GABA_A_R in the striatum. (a) Comparison of the GABA_A_R in different groups, (A) IDPN group, (B) saline group, (C) IDPN+Tia group, and (D) IDPN+JPZDD group. Magnification 20 x. (b) The optical density was quantified. The bar graphs represent data from 24 different animals. Data were shown as mean ± SEM (*n* = 6 mice/group). *※* represents *P* values <0.05; ★ represents *P* values <0.01.

To further investigate the activity and quantity of GABA_A_R in the striatum, GABA_A_R protein expression was assessed by immunohistochemistry (Figure 3(a)). GABA_A_R protein in IDPN group was higher in the IDPN group compared with the saline group (*P* < 0.01). Moreover, IDPN+JPZDD decreased the abundance of GABA, and a notable improvement was observed in JPZDD treated mice compared to the saline group (*P* < 0.05). Tia had the same effect on the expression of GABA_A_R protein in striatum as JPZDD (*P* > 0.05) (Figure 3(b)).

### 3.4. Expression of GABA_A_R mRNA in the Striatum

**Figure 4:**
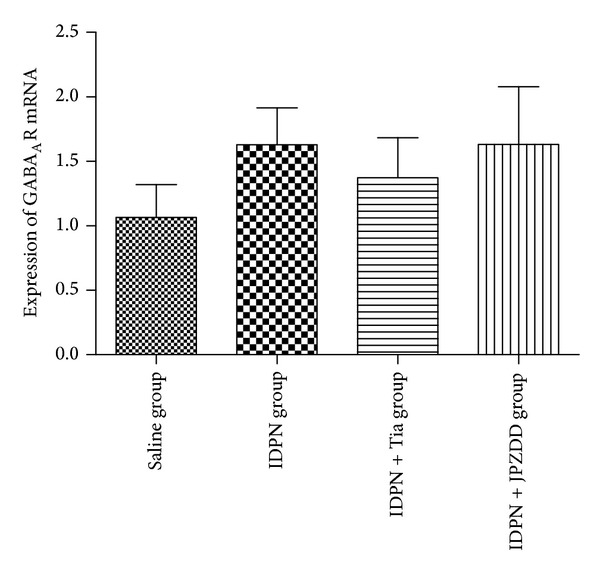
Expression of GABA_A_R mRNA expression in the striatum. The bar graphs represent data from 24 different animals. Data were shown as mean ± SEM (*n* = 6 mice/group).

Real-time quantitative PCR was used to measure the level of GABA_A_R mRNA in mouse striatum. A standard curve was drawn for GABA_A_R alpha 4 and *β*-actin genes. Melting curve analysis confirmed that there were no primer dimers in the PCR products. Results show that GABA_A_R mRNA expression in the IDPN+JPZDD group, IDPN group, and IDPN+Tia group was higher than the control group, but there was no significant difference between these groups (*P* > 0.05) (Figure 4).

## 4. Discussion

In traditional Chinese medicine (TCM), TS is classified as chronic infantile convulsion or tugging and slackening. Its basis pathogenesis is excessive energy of the liver and deficiency of the spleen. Based on this theory, we hypothesized that deficiency of the spleen could cause spleen phlegm and excessive energy of the liver could cause liver wind. The liver wind agitated and spleen phlegm obstructed the channels. According to this hypothesis, the principle of treatment is strengthening the earth (spleen) and suppressing the wood (liver). Under this guide, we created a recipe named JPZDD that included two ancient formulas of Liu-Jun-Zi-Tang (LJZT) and Xie-Qing-Wan (XQW). The main function of XQW is suppressing the liver wind, while LJZT can strengthen the spleen. The herbs in JPZDD were strictly based on the compatibility theory of TCM. The chief drugs in JPZDD are* Pseudostellaria heterophylla* Pax, which nourishes Qi, and* Gentiana scabra* Bge, which purges liver fire to cease liver wind.* Poria cocos* Wolf and* Atractylodes macrocephala* Koidz may invigorate the spleen-qi and* Citrus reticulata* Blanco and* Pinellia ternata* Breit may eliminate the phlegm; these four herbs are adjuvant herbs.* Saposhnikovia divaricata* Schischk and* Uncaria rhynchopylla* Jacks may clear away the liver fire and cease liver wind.* Ligusticum chuanxiong* Hort and* Angelica sinensis* Diels may nourish the liver blood and harmonize the liver wind; these are assistant herbs.

Our previous studies have demonstrated that JPZDD was an effective decoction, which could reduce the times of spontaneous hyperkinesis [21, 22]. At the same time, we have focused on the JPZDD regulation of monoamine neurotransmitters and their metabolites in TS mice. A tenfold dosage was given and satisfactory results were achieved. By IHC and in situ hybridization, we found that JPZDD could increase the expression of DAT protein and mRNA in varying degrees. Its antitic effect might partly be attributed to the synergistic interactions consisting of antioxidation and cognitive improvement [17].

In this study, we chose to use the TS mouse model described by Wang et al. [17]. This model is induced by IDPN (3, 3-iminodipropionitrile). This mouse model displays a series of stereotyped behaviors, such as lateral and vertical head twitching, circling, sniffing, and glomming. These symptoms last at least 2 months without any intervention. Our behavioral studies have further verified the stability of this model. There were no statistical differences in the saline and IDPN groups while there were statistical differences in the INPN + JPZDD and IDPN + Tia groups before and after the treatment. Therefore, we infer that JPZDD could improve the symptoms of TS.

As is well known, many amino transmitters are involved in the CSTC circuits, including GABA and GLU. GABA is the major inhibitory neurotransmitter in mammalian brains. GABA is produced by GABAergic neurons and released at GABAergic synapses formed between GABAergic neurons and their targets [23]. Many GABAergic interneurons of the cerebral cortex migrate tangentially from the same embryonic regions in the ganglionic eminence and give rise to the GABAergic medium spiny projection neurons of the striatum [24]. GABAergic interneurons were shown to play a key role in regulating the development of the cortex, striatum, cerebellum, and hippocampus [25, 26]. Postmortem analysis of TS patients showed decreased numbers of PV positive GABAergic interneurons in striatum and increased numbers in the globus pallidus [27, 28]. Clinical study had also shown that several GABA and Ach-related genes were involved in the pathophysiology of TS and tics [12]. Our experimental results showed that the content of GABA was higher in IDPN, IDPN + Tia, and IDPN + JPZDD groups than in the control group. This result contradicted Lerner's study [14]. We inferred that GABA was produced by glutamic decarboxylation and the relative increase in the Glu activity exerted neurotoxic effect, so the content of GABA increased along with the increase of Glu to keep the new balance. A previous study also showed that high concentrations of Glu increased the extracellular pool of taurine and glycine and then increased extracellular GABA [29, 30].

The rapid inhibitory responses characteristic of GABAergic transmission are mediated by the activation of GABA_A_R receptors [31]. Dysfunction involving the GABA_A_R system may play a major role in the pathophysiology of TS [1]. Baclofen, a synthetic GABA analogue, exerted antispasmodic effects and had been found to benefit children with TS [32, 33]. Therefore, we hypothesized that abnormalities of GABA in TS may be associated with abnormal expression of GABA receptors. To the best of our knowledge, no other study has compared GABA receptors between TS models and controls. Therefore, our study may shed light on the GABAergic mechanism of TS. Our experimental results also revealed that more stereotypy behaviors correlated with higher expression of GABA_A_ receptor, with statistical significance between the saline group and the other groups. The IHC indicated that JPZDD and Tia might decrease the bioactivity of GABA_A_ receptors, so as to maintain balance. Combined with previous results, it is reasonable to postulate that the decrease of GABA_A_ receptor protein expression in striatum which led directly to the altered GABA content was one potential antitic effect of JPZDD. PCR showed no statistical difference between these groups, so the reduction in GABA_A_ receptor was mainly at the protein level rather than at the nucleic acid level. Although there was no difference between the JPZDD group and the Tia group, JPZDD-treated animals did not show adverse side effects, such as extrapyramidal symptoms, tardive dyskinesia, drowsiness, or hyperprolactinemia.

However, the present study has some limitations as follows. First, there are differences between humans and mouse; thus further experiments performed on TS patients can provide more clinical implication for TS treatment. Second, this study did not explain the phenomenon of lower expression of GABA in some patients. Third, other types of GABA receptor could be tested in the future.

In conclusion, results from the study suggested that JPZDD might effectively inhibit stereotype actions and control TS symptoms by downregulating the GABA_A_ receptor expression, which may directly decrease the excessive generation of GABA in the striatum. Thus therapeutic strategies targeting the GABAergic system could be effective in treating TS.
